# The current state of molecular profiling in gastrointestinal malignancies

**DOI:** 10.1186/s13062-022-00322-0

**Published:** 2022-06-06

**Authors:** Reetu Mukherji, Chao Yin, Rumaisa Hameed, Ali Z. Alqahtani, Monika Kulasekaran, Aiwu R. He, Benjamin A. Weinberg, John L. Marshall, Marion L. Hartley, Marcus S. Noel

**Affiliations:** 1 The Ruesch Center for the Cure of GI Cancers, Lombardi Comprehensive Cancer Center, Georgetown University Medical Center, 3800 Reservoir Rd. NW, Washington, DC 20007 USA; 2 MedStar Georgetown University Hospital, 3800 Reservoir Rd. NW, Washington, DC 20007 USA

**Keywords:** Gastrointestinal, GI, Molecular profiling, Next-generation sequencing, NGS, Liquid biopsy, ctDNA, CTC, Gastroesophageal cancer, Colorectal cancer, Pancreatic cancer, Hepatobiliary cancer, Biomarker

## Abstract

This is a review of the current state of molecular profiling in gastrointestinal (GI) cancers and what to expect from this evolving field in the future. Individualized medicine is moving from broad panel testing of numerous genes or gene products in tumor biopsy samples, identifying biomarkers of prognosis and treatment response, to relatively noninvasive liquid biopsy assays, building on what we have learned in our tumor analysis and growing into its own evolving predictive and prognostic subspecialty. Hence, the field of GI precision oncology is exploding, and this review endeavors to summarize where we are now in preparation for the journey ahead.

## Introduction

**Fig. 1:**
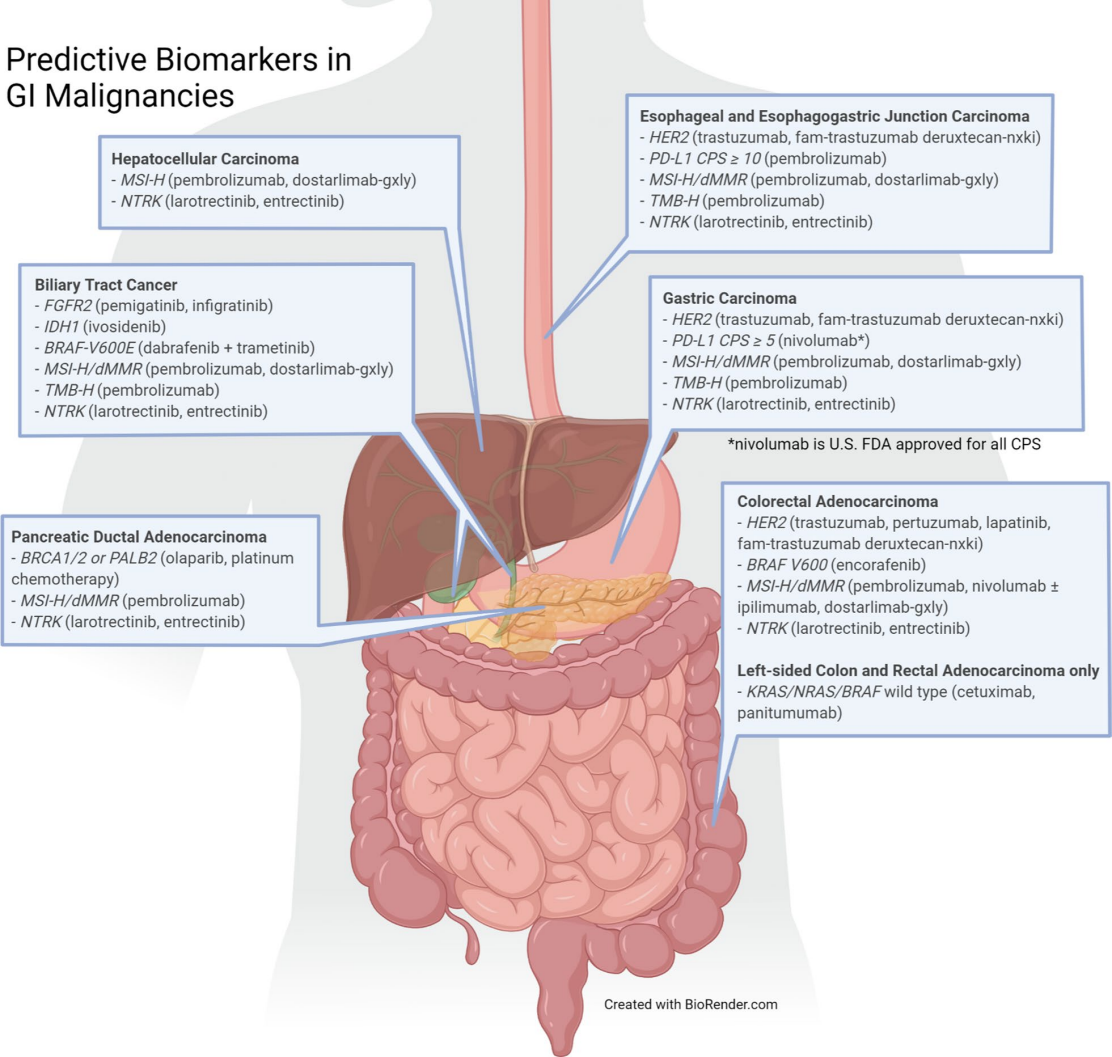
Predictive biomarkers in GI malignancies

Comprehensive molecular profiling has evolved over the last decade. The evolution of molecular profiling changed the face of oncology from standard chemotherapy based on histology to personalized therapy. Using immunohistochemistry (IHC), fluorescence in situ hybridization (FISH), and whole-genome sequencing, oncologists are able to recognize the genomic drivers of tumorigenesis and provide patients with prognostic biomarkers and targeted therapy options (Fig. 1; Table 1).

**Table 1:** Current NCCN predictive markers

Molecular abnormality	Test	Method	When	Details
Colon cancers 2.2021				
MLH1, MSH2, MSH6 or PMS2 mutations	MMR protein expression	IHC	Work-up	Universal testing for MMR or MSI is recommended in all patients newly diagnosed with colorectal cancer. dMMR and MSI-H testing are recommended to predict response to pembrolizumab. Patients with Stage II MSI-H tumors generally have a good prognosis and do not benefit from 5-FU adjuvant therapy.
MSI	MSI; changes in short repeated DNA sequences	PCR NGS	Work-up	
KRAS/NRAS	KRAS (exon 2,3,4) gene; NRAS (exon 2,3,4) gene mutations	NGS	Work-up for metastatic disease: primary tumor and/or metastases	Patients with any known *KRAS* mutation (exon 2,3,4) or *NRAS* mutation (exon 2,3,4) should not be treated with either cetuximab or panitumumab. A *BRAF* V600E mutation makes response to panitumumab or cetuximab highly unlikely unless given with a BRAF inhibitor (e.g., vemurafenib or encorafenib). If patient tumors have a known *RAS/RAF* mutation, *HER2* testing is not indicated. Anti-HER2 therapy is indicated only in *HER2*-amplified tumors that are also *RAS* and *BRAF* wild type.
BRAF	V600E mutation	IHC NGS		
HER2 (ERBB2)	Gene amplification	FISH IHC NGS		
NTRK1/2/3	Gene fusion	FISH IHC RT-PCR NGS	Not specified	TRK inhibitors have activity in patients with *NTRK* fusions (not mutations). Data support limiting testing for *NTRK* fusions to tumors that are *KRAS, NRAS, BRAF,* and *HER2* wild-type and (arguably) those that are MMR deficient (dMMR)/MSI-H.
Gastric/esophageal/GEJ Cancers 2/2021				
HER2	Amplification	(F)ISH NGS	Work-up any time for suspected or documented inoperable locally advanced, recurrent, or metastatic adenocarcinoma	Particularly if trastuzumab therapy is being considered.
	Protein expression	IHC		
PD-L1 (CD274) and HER2 protein	Protein expression	IHC		HER2 negative status corresponds with higher PD-L1 expression rates. Together with MMR, HER2 is a potential biomarker for anti-PD-L1 therapy PD-L1 and MSI testing should be considered on locally advanced, recurrent, or metastatic esophageal or GEJ cancers in patients who are candidates for treatment with PD-1 inhibitors. In gastric cancer, universal testing for MSI/MMR is recommended in all newly diagnosed patients. If sufficient tissue is available, broader NGS may be contemplated. At present, three targeted therapeutic agents have been approved by the FDA for use in esophageal and GEJ cancers: trastuzumab (HER2 positivity), ramucirumab, and pembrolizumab (MSI/MMR, PD-L1 expression, or high tumor mutation burden [TMB; by NGS]). NGS offers the opportunity to assess numerous mutations simultaneously.
MSI	MSI; changes in short repeated DNA sequences	PCR NGS		
MLH1, MSH2, MSH6 or PMS2 mutations	MMR protein expression	IHC		
NTRK1/2/3	Gene fusion	NGS	Not specified	The FDA approved the use of select TRK inhibitors (entrectinib, larotrectinib) for NTRK gene fusion-positive solid tumors.
Hepatobiliary cancer 1.2021				
MSI	MSI; changes in short repeated DNA sequences	PCR	Prior to primary treatment of metastatic or unresectable Gallbladder Cancer or metastatic intrahepatic cholangiocarcinoma	The PD-1 inhibitor, pembrolizumab can be used in patients with MSI-H/dMMR/TMB-H tumors.
MLH1, MSH2, MSH6 or PMS2 mutations	MMR protein expression	IHC		
FGFR2	Gene fusion	NGS	Evaluate for subsequent lines of therapy for unresectable or metastatic bile duct cancer	FGFR2 inhibitors (pemigatinib, infigratinib) are an option for cholangiocarcinoma with FGFR2 fusions or rearrangement.
IDH1	Mutation	NGS	Evaluate for subsequent lines of therapy for unresectable or metastatic bile duct cancer	IDH1 inhibitor (ivosidenib) can be used for cholangiocarcinoma with IDH1 mutations.
BRAF	V600E mutation	NGS	Evaluate for subsequent lines of therapy for unresectable or metastatic bile duct cancer	Dabrafenib + trametinib can be used for BRAF V600E mutant tumors.
NTRK1/2/3	Gene fusion	FISH RT-PCR NGS	Hepatobiliary cancer	TRK inhibitors (entrectinib, larotrectinib) have activity in patients with NTRK fusions.
Pancreatic 2/2021				
*BRCA1* & *BRCA2* *BRAF* *HER2* *KRAS* *PALB2*	Mutation (somatic and germline)	IHC NGS	Initial work-up Tumor and blood	Cell-free DNA testing can be considered if tumor tissue testing is not feasible. 9% of pancreatic cancers harbor a germline or somatic *BRCA1* or *BRCA2* mutation. Olaparib can be used if patient disease has not progressed on first-line platinum-based chemotherapy. Other DDR enzyme inhibitors may be effective. An EGFR inhibitor (e.g., erlotinib) = chemotherapy may benefit *KRAS*wt patients. A *BRAF* mutation makes this response unlikely.
Fusions: inc ALK, NRG1, NTRK, ROS1		IHC PCR NGS		Fusions are rare but, taking NTRK as an example, TRK inhibitors (e.g., larotrectinib) in these patients very effective. Other fusion inhibitors are experimental in pancreatic cancer but an option.
MLH1, MSH2, MSH6 or PMS2 mutations	MMR protein expression	IHC	Work-up for locally advanced or metastatic disease	Pembrolizumab is an option in first-line and beyond, particularly for patients with MSI-H and dMMR tumors and no other satisfactory treatment options.
MSI	MSI; changes in short repeated DNA sequences	PCR NGS		

Most biomarkers in these tables are classed as “useful in certain circumstances”

Testing in a CLIA-approved laboratory using validated tests or panels is recommended. For NGS, a CLIA-approved *high-complexity* laboratory is recommended

NGS can pick up rare and actionable mutations and fusions and is recommended for all GI cancers

Molecular profiling using tissue next-generation sequencing (NGS) has become a standard of care practice, and recently, circulating tumor DNA (ctDNA) has emerged as a tool for molecular profiling, a predictor of response to systemic treatment, and a powerful way to measure minimal residual disease (MRD) using less invasive approaches [1–3].

A decade ago, the treatment of colorectal cancer (CRC) was the first of all GI malignancies to be influenced by molecular profiling. It was initially observed that patients with *KRAS* mutant CRC do not respond to epidermal growth factor rector (EGFR)-targeted agents such as the monoclonal antibodies cetuximab and panitumumab [4, 5]. Later, it was recognized that *BRAF*, *NRAS*, and *PIK3CA* mutations and *HER2* mutations and amplifications also confer non-responsiveness to EGFR-targeted agents and carry a generally poorer patient prognosis than wild-type disease [6, 7]. Subsequently, patients with *BRAF* V600E mutant CRC were shown to benefit from treatment with vemurafenib, a small molecule tyrosine kinase inhibitor [8], and later, encorafenib combined with cetuximab emerged as a standard of care for this subset of patients following chemotherapy [9] (Table 1). With the advent of immune checkpoint inhibitors (ICIs) that target PD-1/PD-L1, microsatellite instability (MSI) was found to be the most significant predictor of CRC treatment response. MSI can be sporadic or driven by germline mutations in one of the MMR genes (MLH1, MSH2, MSH6, or PMS2), as found in hereditary Lynch syndrome [10, 11].

MSI testing is essential for all GI malignancies because localized MSI-high (MSI-H) tumors will have a good prognosis, and advanced disease will likely respond to PD-1/PD-L1 inhibitors [12, 13] (Table 1). Until very recently, advanced gastric and gastroesophageal cancers were treated solely with conventional chemotherapy. Now, advances in molecular profiling and signaling pathway knowledge have provided new treatment options for patients with PD-L1 or HER-2 overexpressing tumors, including PD-1/PD-L1 inhibitor therapy, HER2 targeted treatment, and anti-vascular endothelial growth factor (VEGF) antibody therapy [14–17].

Advances in molecular profiling have led to therapeutic options targeting advanced biliary tract cancers with IDH1/2 mutations, FGFR alterations, HER2 amplifications, and *BRAF* V600E mutations [18–21] (Table 1).

In pancreatic cancer, understanding the role of germline testing for *BRCA1*, *BRCA2*, and *PALB2* in homologous recombination repair has allowed the emergence of poly(adenosine diphosphate-ribose) polymerase inhibitors (PARPi) as a treatment option [22].

## Biomarkers

### PD-L1

PD-1 is an inhibitory receptor expressed on several immune cells, particularly cytotoxic T cells. It interacts with 2 ligands: PD-L1 and PD-L2. PD-L1 is expressed on tumor cells and immune cells, whereas PD-L2 is expressed on macrophages and dendritic cells. The interaction of PD-1 with PD-L1 inhibits T-cell activation and cytokine production, which is vital to maintaining homeostasis of the immune response and preventing autoimmunity [23, 24]. However, PD-1/PD-L1 interactions within the tumor microenvironment provide an immune escape pathway for tumor cells by turning off cytotoxic T cells [25]. Tumor cells upregulate the PD-1 receptor or ligand to evade destruction by the host immune system. Thus, in blocking the PD-1 pathway with antibodies to PD-1 and PD-L1/PD-L2, the adaptive immune response is activated against tumor cells resulting in an anticancer response. Tumor cell PD-L1 expression is associated with response to anti-PD-1/anti-PD-L1 therapy [26].

PD-L1 protein expression in many cancer types, assessed via immunohistochemistry (IHC), is one of the FDA-approved predictive biomarkers for anti-PD-1 and anti-PD-L1 ICI monotherapy [26]. However, PD-L1 expression within tumors and between tumor sites may be heterogeneous [27–29], and assays may give variable results. To the latter point, there are multiple qualitative PD-L1 assays involving different antibodies to assess the expression of PD-L1 by IHC using chromogenic methods [30], and different antibody assays may give different results.

In esophageal/GEJ/gastric cancer, the PD-L1 combined positive score (CPS) has been tested as a predictive biomarker for immunotherapy. CPS is the number of cells staining for PD-L1 cells (tumor cells, lymphocytes, and macrophages) divided by the total number of evaluated tumor cells, multiplied by 100 [31]. Tumors are considered PD-L1 positive if they have a CPS > 1. A positive CPS is associated with improved GI cancer patient outcomes upon ICI therapy. In KEYNOTE 062, KEYNOTE 061, and KEYNOTE 059, the PD-1 inhibitor pembrolizumab demonstrated efficacy against gastric and GEJ cancer as first, second, or third-line treatment based on a CPS of > 1 [32]. On the other hand, the PD-1 inhibitor nivolumab is FDA approved in esophageal/GEJ/gastric cancers regardless of CPS, based on CHECKMATE 648 and CHECKMATE 649 studies [14, 33]. How to use CPS in the selection of upper GI cancer patients for frontline ICI therapy remains a point of debate in the oncology community [34].

### TMB: tumor mutation burden

TMB, defined as the total number of exon mutations in a tissue sample [35], has emerged as an important biomarker associated with immunotherapy response in multiple tumor types [26]. TMB is a critical driver in the generation of immunogenic neopeptides presented on major histocompatibility complexes on the tumor cell surface [36]. These immunogenic components influence response to ICIs, meaning that TMB impacts ICI efficacy. This TMB effect on ICI efficacy is reflected in many retrospective studies, including the Phase II Keynote-158 trial [37], which led to the FDA approval of pembrolizumab in patients with unresectable or metastatic high TMB (≥ 10 mutations/megabase) solid tumors [38].

Detecting ctDNA in the blood is a noninvasive test called liquid biopsy (see the section on Molecular profiling in the blood). With an increasing interest in ctDNA, studies have been carried out to develop methods, including NGS, that can estimate the tumor fraction in a patient’s plasma and measure the TMB from their blood with high accuracy [39]. Chalmers et al. [40] demonstrated that TMB can be accurately measured in blood by sequencing targeted gene panels, but accuracy is compromised when the sequenced genome region is less than 0.5 MB. The Guardant Health Omni panel (500 genes, 2.1 MB) and Foundation Medicine bTMB panel (394 genes, 1.14 MB) are plasma-based NGS assays containing sufficiently large genome region sizes to measure TMB across a broad range of TMB values. In a study by Qiu et al. [41], Guardant Health and Foundation Medicine tests were evaluated and compared in their ability to evaluate TMB from ctDNA. The investigators ascertained that tissue and plasma TMB correlated well using both assays as long as analyzed samples contained a high TMB; the correlation was compromised if samples contained only low to medium TMB [41].

**Fig. 2:**
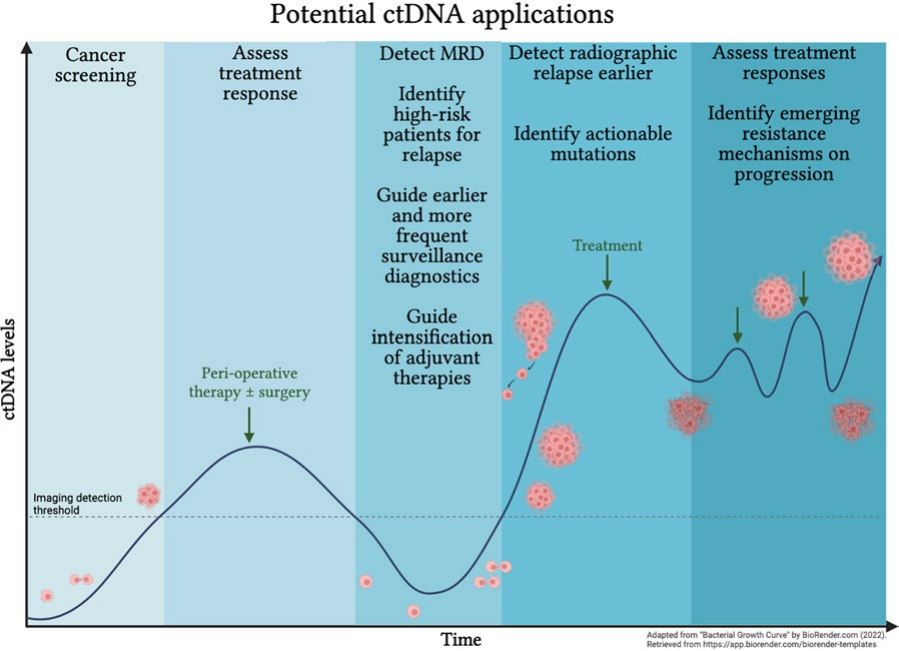
Potential ctDNA applications

TMB has been used as a predictor for response to ICI therapy, and early measurements of ctDNA were shown to help detect treatment failure [42]. MRD is another important biomarker of treatment failure. It refers to residual tumor cells present after cancer treatment and is associated with disease recurrence. MRD can be detected in blood using techniques like quantitative PCR and NGS. However, most recently, ctDNA has been used to detect MRD in the blood, serving as a powerful diagnostic and predictive tool (Fig. 2) [43].

### MSI

Microsatellite instability (MSI) is a molecular fingerprint for defects in the mismatch repair system (dMMR), which is associated with an increased risk of cancer [44]. The MMR system is composed of heterodimers (MSH2/MSH6 and MSH2/MSH3 complexes) that ensure the specific recognition of mispaired nucleotides generated due to DNA damage [45]. In humans, these complexes initiate DNA repair and recruit MLH1/PMS1, MLH1/PMS2, and MLH1/MLH3 heterodimers to catalyze the excision of the mispaired nucleotides as well as error-free DNA resynthesis. Genetic and epigenetic inactivation of MMR genes cause MMR defects (dMMR) and give rise to spontaneous, genome-wide mutations [45]. This mainly affects the short tandem repeat DNA sequences termed microsatellites, which occur at specific foci throughout the genome.

MSI-H tumors contain many mutation-associated neoantigens, which, it is believed, are recognized as foreign by the immune system. The benefit of ICIs for patients with MSI-H/dMMR tumors was first documented in a Phase II trial, in which patients with metastatic cancer (78% colorectal) with and without dMMR received pembrolizumab. Only the patients with MSI-H/dMMR tumors benefited from the ICI therapy [46]. Results from this trial were confirmed in the larger Phase II KEYNOTE 158 study evaluating pembrolizumab in dMMR metastatic colorectal patients [37]. Later, results from the KEYNOTE 177 trial of pembrolizumab as first-line treatment for patients with MSI-H/dMMR metastatic colorectal cancer showed longer progression-free survival (PFS) compared to standard chemotherapy [47]. Similarly, the CHECKMATE 142 trial suggested durable benefit from combined nivolumab plus ipilimumab (anti-CTLA-4) in patients with MSI-H/dMMR tumors [48].

Conventional methods used for MSI testing include immunohistochemistry (IHC) and PCR-based assays performed on tumor tissue samples. Tumor tissue-based NGS can also determine MSI status.

### HER2

HER2 is a member of the human epidermal growth factor receptor (HER) family. This family includes HER1 (ErbB1; EGFR), HER2 (ErbB2), HER3 (ErbB3), and HER4 (ErbB4) [49–52]. The HER2 receptor regulates normal cell proliferation, survival, and differentiation via different signal transduction pathways. Amplification or overexpression of *HER2* is found in 2–6% of patients with metastatic colorectal cancer [53–55]. The frequency of *HER2* overexpression in gastric and gastroesophageal cancer ranges from 4.4 to 53.4%, with a mean of 17.9% [56, 57].

Several strategies have been developed to target HER2, including extracellular antibodies like trastuzumab, which targets domain IV of the receptor, and pertuzumab which binds to domain II and inhibits the heterodimerization of HER2 with other ErbB receptors [58]. Additionally, small tyrosine kinase inhibitors like lapatinib, tucatinib, or neratinib inhibit HER2 activity, while antibody–drug conjugates (ADCs), such as trastuzumab emtansine (T-DM1) and trastuzumab deruxtecan (T-Dxd), bind HER2 and introduce a potent cytotoxic agent into cells overexpressing the receptor [16].

The Phase III ToGA (Trastuzumab for Gastric Cancer) trial for patients with gastric or GEJ cancer with overexpression of *HER2* or gene amplification was the first study to demonstrate the therapeutic benefit of targeting HER2 in GI cancers [15]. The US FDA subsequently approved trastuzumab for the first-line treatment of patients with metastatic, *HER2*-positive gastric or gastroesophageal cancer.

Subsequent trials in patients with GI cancers include the Phase 2 HERACLES trial [59] and the ongoing MyPathway basket trial [60]. In the HERACLES trial [59], standard treatment-refractory patients with *KRAS* wild-type (wt) CRC harboring a *HER2* amplification received trastuzumab and lapatinib. In a subset of the ongoing MyPathway basket trial [60], patients with *HER2*-amplified metastatic CRC received pertuzumab plus trastuzumab. The objective response rates were around 30% in both studies, and several other patients had stable disease, demonstrating that *HER2* amplification is an actionable target. More recently, a trial of T-Dxd in previously treated patients with gastric or GEJ cancer found improved overall survival (OS; 12.5 months vs. 8.4 months; *P* = 0.0097) and ORR (40.5% vs. 11.3%) compared to standard chemotherapy. These results led to the US FDA approval of T-Dxd in the third or later lines of therapy [16].

The development of tumor resistance to HER2 inhibitors is a problem for which there are multiple possible mechanisms, including loss of *HER2* expression and HER3 ligand-dependent HER2-HER3 interactions leading to evasion of apoptosis [61]. A TAF/FGF5/FGFR2/c-Src/HER2 axis might act as a HER2-targeted therapy escape pathway, which seems to be reversed by FGFR inhibition [62].

Prior attempts to demonstrate an OS benefit from second-line HER2-targeted vs. standard cytotoxic therapy have failed, possibly due to loss of HER2 expression following trastuzumab-based first-line treatment. MOUNTAINEER-02 trial investigators hope that dual targeting of HER2 with tucatinib and trastuzumab will overcome this resistance. Tucatinib is a small molecule tyrosine kinase inhibitor, which was shown to have “very potent,” selective activity against HER2, with minimal off-target effects [63]. The ongoing Phase II/III MOUNTAINEER-02 trial [NCT04499924] [64] is enrolling patients with advanced or metastatic *HER2*-positive (overexpression or amplification) gastric or GEJ cancer with disease progression (PD) after frontline therapy, including a HER2-directed antibody. Patients receive second-line treatment with paclitaxel plus ramucirumab, either with tucatinib plus trastuzumab, tucatinib plus trastuzumab-placebo, tucatinib-placebo plus trastuzumab, or two placebos.

Future directions include using liquid biopsy genotyping assays as a viable, real-time alternative to tissue-based genotyping in the identification of HER2 alterations in the metastatic setting. HER2 copy number is typically assessed using surgically-obtained tissue, but necessary information can now be obtained conveniently and noninvasively using ctDNA (See the “Molecular profiling in blood” section).

### FGFR 1–4

Fibroblast growth factors (FGFs) and their receptors (FGFR 1, 2, 3, and 4) are vital to many cellular processes. After ligand stimulation, FGFRs undergo dimerization and phosphorylation, prompting intracellular signaling that triggers a number of intracellular survival and proliferative pathways [65–67]. Aberrant FGFR signaling (found in just over 7% of all cancers) has been shown to have an oncogenic role. FGFR alterations (primarily in FGFR2) are found in approximately 13% of intrahepatic cholangiocarcinomas (CCA), 3% of gallbladder cancers, 9% of gastric cancers in a Western population, and 3% of gastric cancers in an Asian population) [68–73].

Of all *FGFR2* aberrations, 66% are amplifications, 26% mutations, and 8% rearrangements [74]. Oncogenesis most often occurs through FGFR pathway activation. For example, FGFR amplifications and rearrangements lead to protein overexpression and dependence on FGFR signaling, although conversely, preclinical models suggest that amplifications also predict increased sensitivity to FGFR inhibition [75, 76]. Mutations in *FGFR*s cause increased downstream phosphorylation [72, 75].

FGFR has become a molecular target of increasing interest in CCA. There are several FGFR-targeted therapies of interest, mostly in the form of tyrosine kinase inhibitors (TKIs). Pemigatinib is a selective oral FGFR1-3 inhibitor investigated in the open-label single-arm FIGHT-202 trial for previously treated advanced CCA [77]. Among patients with FGFR2 alterations, pemigatinib displayed an overall response rate (ORR) of 36%, disease control rate (DCR) of 80%, and median duration of response (DOR) of 7.5 months. Pemigatininb is now FDA approved for previously treated, unresectable, locally advanced, or metastatic CCA with an FGFR2 alteration. Pemigatininb combined with gemcitabine and cisplatin is currently being studied in the first-line, phase III FIGHT-302 trial (NCT03656536). Infigratinib, another selective FGFR 1–3 TKI, obtained accelerated FDA approval in subsequent-line settings for FGFR2-altered CCA. This drug demonstrated an ORR of 23% and mDOR of 5 months [78]. In CCA, other drugs currently under study include derazantinib (FGFR1-3 inhibitor) and erdafinitib (FGFR1-4 inhibitor). Toxicities of FGFR inhibitors are predictable and similar across this class of therapeutics and include hyperphosphatemia (50–80%), nail toxicity (35%), and ophthalmologic toxicity (4–9%) [77, 79–81].

### IDH1/2

Isocitrate dehydrogenase (IDH) is a key enzyme in the tricarboxylic acid cycle and comprises 2 subtypes: IDH1, located in the peroxisomes and cytosol, and IDH2, located in the mitochondria [82, 83]. In CCA, *IDH1* mutations are found in 15–25% of cases, particularly in intrahepatic CCA. *IDH2* mutations are less frequent, found in up to 3% of CCAs [84, 85]. *IDH* mutations generally lead to a gain-of-function that disrupts normal catalytic activity. The net effect is increased conversion of α-ketoglutarate to D-2-hydroxyglutarate, which leads to downstream cellular proliferation through pathways including DNA methylation and VEGFR [82, 83, 86].

Multiple IDH-selective inhibitors are being investigated in vitro and in clinical trials. Ivosidenib, an oral small-molecule inhibitor, was among the first to be studied clinically: A phase I study confirmed tolerability and demonstrated a median PFS (mPFS) of 3.8 months in previously treated patients with *IDH1*-mutated CCA [87]. The recent ClarIDHy Phase III placebo-controlled trial demonstrated an OS benefit from ivosidenib that trended towards statistical significance (mOS 10.3 vs. 7.5 months, HR 0.79, 95% CI 0.56–1.12; *p* = 0.093), becoming statistically significant once a mathematical model adjusting for treatment crossover effects was employed (mOS 10.3 vs. 5.1 months, HR = 0.49; 95% CI 0.34–0.70; *p* < 0.0001) [88]. Ivosidenib was granted FDA approval in August 2021 [89]. Additional promising IDH1-targeting drugs are under early investigation, including another small-molecule inhibitor, olutasidenib (NCT03684811).

### BRCA/PALB2

Germline mutations in *BRCA1* and *BRCA2* are well studied and associated with a high risk of cancer, particularly breast and ovarian cancers, with high hereditary penetrance in an autosomal dominant pattern [90–92]. This associated risk has been established across other cancers, including pancreatic cancer, where *BRCA2* mutations pose a relative risk of 3.5–10 compared to non-carriers.

*BRCA1* and *BRCA2* are suppressor genes of the same family, located on long arms of chromosome 17 and 13, respectively [93, 94]. They play instrumental roles in DNA damage response, particularly in maintaining chromosomal stability in the process of homologous recombination repair [95, 96]. One of the early successes of whole-genome sequencing was the identification of the *BRCA2* and its partner and localizer gene *PALB2* in 1–4% of familial PDAC [90, 97, 98]. *PALB2* colocalizes with *BRCA2* at the site of DNA damage to enable DNA repair [99].

Deficiencies in homologous recombination and DNA repair pathways predict sensitivity to platinum-based chemotherapy regimens as well as poly(ADP-ribose) polymerase inhibitors (PARPi). Patients with PDAC and homologous recombination gene mutations had improved PFS and OS when treated with frontline platinum-based therapy compared to patients without such mutations (HR 0.44, 95% CI 0.29–0.67; *P* < 0.01) [100, 101]. Similarly, PARPi appear to be active in PDAC; the phase III POLO trial demonstrated sensitivity to olaparib of patient tumors with homologous recombination gene mutations. Maintenance olaparib after platinum-based induction therapy showed superior mPFS to placebo (7.4 vs. 3.8 months, HR 0.53, 95% CI 0.35–0.82) [102]. Olaparib is FDA-approved in the maintenance setting for PDAC.

### BRAF V600E mutation

The *BRAF* V600E mutation is found in about 8–10% of CRCs [103] and 3–7% of bile duct cancers [71].

In the NCI-MATCH EAY131-H trial, a combination of dabrafenib (BRAF inhibitor) and trametinib (MEK inhibitor) produced favorable response rates in a total of 35 pretreated patients with a range of solid tumors, all harboring a BRAF V600E mutation [104]. A confirmed objective response rate (ORR) of 37.9% (90% CI 22.9–54.9%; P < 0.0001 against a null rate of 5%) was reported. The median duration of response was 25.1 months. Four of the 35 patients enrolled on trial had CCA, and 3 of these 4 experienced a partial response (PR) [104].

The same drug combination was tested in patients with bile duct cancer harboring BRAFV600E mutations as part of the ROAR Basket Trial [21]. The ROAR Basket Trial enrolled 9 different cohorts of 178 patients with rare malignancies, all harboring BRAF V600E mutations. The bile-duct cancer cohort included 35 patients treated with the combination of the BRAF inhibitor dabrafenib and the MEK inhibitor trametinib. Most patients (74%) had stage IV disease at enrollment, and all 35 patients with biliary tract cancer had received prior chemotherapy (80% had received at least 2 prior lines). The median duration of treatment exposure was 6 months, with 86% of the patients being treated for more than 3 months. The median follow-up was 8 months. A PR was reported in 42% of the cohort by investigator assessment and 36% by independent review. Stable disease was achieved in 45% and 39%, respectively, and 12% had progressive disease as their best response (by either assessment method). The mPFS by investigator assessment was 9.2 months, and the median OS was 11.7 months. With regard to safety, adverse effects (AEs) were found to be comparable to those previously reported with each agent alone, with no new toxicities observed. Potential study treatment-related toxicities included fatigue, neutropenia, hyponatremia, and hypophosphatemia. No grade 5 events were observed [21].

## Molecular profiling in the blood

A liquid biopsy identifies components of a tumor in the blood, such as ctDNA, circulating tumor cells (CTC), circulating tumor RNAs, and circulating tumor exosomes [105]. Of all these entities, ctDNA is the most studied to date and comprises fragments of cell-free DNA (cfDNA) that retain tumor-specific mutations and epigenetic characteristics [106–108]. These fragments are released into the circulation spontaneously or after apoptosis or necrosis. ctDNA makes up a highly variable fraction of total cfDNA in peripheral blood, and this fraction (reportedly ranging from less than 0.1% to almost 90%) is impacted by disease stage, tumor type, and analysis technique, to name a few. The half-life of ctDNA is 16 min to 2 h, allowing for indirect real-time tumor characterization [109–111]. Although the liquid biopsy was described as early as the 1940s, it is only recently, with enhancements in genomic sequencing techniques and identification of novel biomarkers, that we have seen more commercialized applications of liquid biopsy platforms [112–114].

### Overview

The advantages of blood-based molecular profiling include the ability to study intra- and inter-tumor genomic heterogeneity, the ability to perform tumor profiling in the absence of available tissue, avoidance of invasive biopsy procedures, the feasibility of frequent longitudinal testing, and a quick turnaround time to inform treatment plans [115]. To date, numerous commercial liquid biopsy profiling assays are available to providers. Methodologies overlap with tissue profiling and involve comprehensive genomic, single-gene, or hotspot gene testing. In hotspot testing, commonly altered regions within select genes are evaluated. Of the comprehensive genomic tests, only two are FDA approved today. Guardant Health’s Guardant360 CDx, targeting 55–74 genes using DNA NGS, was first FDA approved on August 7, 2020. Roche’s FoundationOne Liquid CDx, targeting over 300 genes using DNA NGS, was first approved on August 26, 2020 [116–118]. Although approved as companion diagnostic tests for therapeutics in lung, prostate, ovarian, and breast cancer, these assays are increasingly studied and adopted in GI cancers to identify prognostic and predictive biomarkers, actionable mutations with FDA-approved therapies, responses to treatment, and mechanisms of resistance. These and other comprehensive gene-based assays utilize NGS and can identify single nucleotide variants (SNVs), insertion and deletions, gene rearrangements, copy number variants (CNVs), TMB scoring, and microsatellite status [112, 116, 119, 120].

Numerous studies have recently evaluated ctDNA genomic profiling in GI cancers to identify actionable mutations, monitor disease response, and understand resistance mechanisms (Fig. 2; Table 2). Concordance rates of 85–98% have been reported for ctDNA and tissue genomic profiling, and promising sensitivities and specificities in NGS/PCR-based ctDNA assays, for example, 50–93% and 97–100% respectively for *RAS* mutations, 63–100%, and 98–100% for *BRAF* mutations, and 33–98% and 98% for *ERBB2* amplifications, have been observed [109, 121–123]. In addition, studies have suggested that ctDNA captures genomic heterogeneity between primary and metastatic sites, which should act to enhance therapy selection [124]. As a noninvasive and convenient test, blood-based genomic profiling has the potential to replace or complement tissue testing, particularly when considering targeted therapies.

**Table 2:** Select Examples ctDNA studies in GI cancers

Gene	Tumor	Study type and details	Sample #	Assay utilized	Findings	Reference
*RAS*	CRC	Prospective cohort	98 pts	BEAMing expanded *RAS* mutation panel	*RAS* mut status was evaluable in plasma *RAS* plasma-tissue concordance was 91.8% ctDNA MAF was associated with clinical stage *RAS* testing using BEAMing was comparable to tissue testing	[125]
*RAS*	mCRC	Prospective	236 pts	OncoBEAM	*RAS* plasma-tissue concordance was 92% Plasma false-negatives were more frequent in lung-metastases-only disease	[126]
*RAS*	mCRC	Prospective	280 pts	OncoBEAM	*RAS* plasma-tissue concordance was 86.4%, positive percent agreement was 82.1%, and negative percent agreement was 90.4% Lung-metastases-only disease was associated with more discordance (concordance rate of 64.5%), but concordance improved with larger tumor burden	[127]
*RAS/EGFR/BRAF*	mCRC	Retrospective cohort Plasma samples from *RAS/EGFR/BRAF* wt mCRC pts analyzed after PD on anti-*EGFR* therapy	135 pts 496 pts in the validation cohort	Guardant 360 NGS	*RAS* and *EGFR* mut clones decrease exponentially from the time of *EGFR*i discontinuation Confirmed ECD *EGFR* mutation as a potential driver for *EGFR*i resistance Identified half-life of *RAS/EGFR* clones to guide the timing of re-challenge to therapy	[128]
*RAS/EGFR/BRAF*	mCRC	Phase II *EGFR*i therapy rechallenge after prior PD guided by *RAS/BRAF/EGFR* status in ctDNA	27 pts	ddPCR and NGS	69% of screened pts were wt First interventional trial of liquid biopsy—ctDNA molecular selection—driving *EGFR*i rechallenge in mCRC *RAS/BRAF/EGFR* wt pts rechallenged to *EGFR*i demonstrated 30% ORR, 40% SD, 59% DCR with SD > 4 months, mPFS 16 weeks	[129] CHRONOS
*RAS/BRAF*	mCRC	Prospective	72 pts	Idylla Biocartis	*RAS/BRAF* plasma-tissue concordance was 81.94%, with higher concordance in liver metastases cases Emerging *KRAS* mutations were identified in 33% of pts treated with *EGFR*i	[130]
*RAS/BRAF*	mCRC	Prospective Plasma mutational testing prospective series	278 pts	OncoBEAM	*RAS/BRAF* plasma-tissue concordance in chemotherapy naïve pts with liver metastases was 91.8% Supports ctDNA as a surrogate marker to tissue testing for *RAS* and *BRAF* status	[131] ColoBEAM
*RAS/BRAF*	mCRC	Prospective, non-randomized *EGFR*i therapy rechallenge after prior PD guided by RAS/BRAF status in ctDNA	22 pts	PyroMark Q24 MDx Workstation, Genetic Analyzer ABI3130, Idylla RT-PCR, QZ200 System ddPCR	70% of enrolled pts were wt Wt pts who underwent rechallenge experienced 27% ORR, 55% DCR, 7-month mOS, and 3-month mPFS Rechallenge strategy is feasible with molecular selection through ctDNA	[132]
*RAS*	mCRC	Prospective, phase II, single-arm *EGFR*i therapy rechallenge in 3rd line setting after prior PD	28 pts	ddPCR and Ion Torrent S5 XL ultra-deep NGS	48% samples at rechallenge baseline were *RAS* mut wt patients experienced 21% ORR, 32% SD, 54% DCR, mPFS 3.4 mo, and mOS 9.8 mo ctDNA *RAS* status predicted for responses to *EGFR*i. *RAS* wt was associated with a longer mPFS compared to *RAS* mut (4.0 vs. 1.9 mo, *p* = 0.03), with a trend toward longer mOS (12.5 vs. 5.2 mo, *p* = 0.24)	[133] CRICKET
*RAS*	mCRC	Retrospective post-hoc biomarker study (pts from JACCRO CC-08 and 09)	16 pts	OncoBEAM Ras CRC	38% pts at rechallenge were *RAS* mut DCR was better in *RAS* wt compared to *RAS* mut pts (80% vs. 33%, respectively) mPFS in *RAS* mut vs. *RAS* wt pts was 2.3 vs. 4.7 mo (*p* = 0.0042) and mOS 3.7 vs 16 mo (*p* = 0.0002), respectively ctDNA *RAS* status was significantly associated with clinical outcomes in pts receiving *EGFR*i rechallenge	[134]
*BRAF*	mCRC	Retrospective	64 pts	MD Anderson/GuardantHealth LB70 NGS	*BRAF V600E* plasma-tissue concordance was 80% Lower *BRAF V600E* VAF was associated with acquired resistance to *EGFR*i ctDNA to detect *BRAF V600E* mut is feasible	[135]
Multiple genes	mGEC	Cohort study	26 pts 28 pts	Guardant 360	Demonstrated genomic heterogeneity within the primary tumor and in disseminated disease Found discordance between primary tumor and metastases in 36% of patients and high concordance between metastases and ctDNA (85%) ctDNA profiling may enhance selection of therapy by identifying heterogeneous mutation profiles	[124]
*HER2*	GC	Prospective Biomarker study in pts treated with neoadjuvant capecitabine + oxaliplatin + lapatinib in HER2 + GC	32 pts	Guardant 360	Plasma *ERBB2* amplification predicted for chemotherapy + lapatinib responses Changes in plasma *ERBB2* copy number were associated with responses to therapy Plasma genomics at the time of PD revealed emergence of *MYC*, *EGFR*, *FGFR2*, and *MET* amplification Targeting *MET* kinase alongside *HER2* in PDX model tumor that progressed on afatinib and had *MET* amplification resulted in tumor regression	[136]
*HER2*	mGEC	Biomarker analysis from phase II (NCT02954536) *HER2* positive mGEC treated with trastuzumab and chemotherapy in first-line setting	25 pts	Guardant 360 NGS	Baseline ctDNA *ERBB2* amp and decreasing VAF on treatment were predictive biomarkers for response to *HER2*-directed therapy	[137]
*HER2*	mGC	Biomarker analysis from DESTINY-Gastric01 mGC pts treated with T-DXd	151 pts	GuardantOMNI	ORR in pts with baseline plasma *ERBB2* amp was 60.6% and in pts without amp was 34.2% ORR in pts with baseline plasma *ERBB2* copy number above 6 had ORR 75.8% compared to 40.8% in those below 6.0	[138]
*HER2*	mCRC	Prospective, phase II Pertuzumab plus trastuzumab in mCRC (refractory/intolerant to chemotherapy and *EGFR*i) with *RAS* wt and *HER2* amp by tumor or ctDNA analysis	30 pts	Guardant 360 NGS	ORR was 30% in tissue *HER2* amp pts and 28% in ctDNA *HER2* amp pts Pts with tissue + /ctDNA– *HER2* amp had significantly lower ctDNA fraction compared to tissue + /ctDNA + *HER2* amp pts, likely due to low tumor shedding Baseline alterations in resistance pathways *RTK/RAS/PI3K* were enriched in non-responders and more frequently identified by ctDNA (67%) compared to tissue (19%) testing Baseline ctDNA profiles predicted those who would benefit from pertuzumab plus trastuzumab Decreasing ctDNA fraction on tx was associated with superior PFS and radiographic response ctDNA identified an actionable new alteration in 62% of pts after PD	[139] TRIUMPH
*FGFR2*	mGC	Retrospective	365 pts	Guardant 360 NGS and Illumina NextSeq 550	*FGFR2* amp were detected more frequently with ctDNA than with tissue analysis (7.7% vs. 2.6–4.4%, respectively) 2 pts with *FGFR2* amp by ctDNA after PD but not on pretreatment tissue analysis responded to FGFR inhibitors	[140]
*FGFR2*	CCA	Biomarker analysis from 3 pts enrolled in phase II study with infigratinib in *FGFR* mut CCA	3 pts	Guardant 360 NGS	ctDNA testing at the time of progression on infigratinib revealed new *FGFR2* point mutations (resistance mechanisms) ctDNA can reveal heterogeneous concurrent resistance mutations, unlike individual tissue biopsies	[141]
*FGFR2*	CCA	Retrospective	137 pts	Tempus xF liquid biopsy	ctDNA identified more actionable mutations in liquid biopsies (33.1%) compared to tissue biopsies (23.2%) Prevalence of *FGFR2* fusions was higher in liquid biopsies (11.3%) than tissue biopsies (3.4%) ctDNA may be used to guide therapy selection	[142]
Multiple genes	CCA	Prospective	24 pts	15-gene and 710 gene oncopanel	Plasma-tissue concordance was 74% (higher at 94% in intrahepatic CCA and lower at 55% in extrahepatic CCA) Baseline ctDNA VAF correlated with initial tumor loads Baseline ctDNA VAF correlated with PFS in intrahepatic CCA	[143]
*IDH1*	CCA	Biomarker analysis from ClarIDHy	210 samples	BEAMing digital PCR test	*IDH1* mut plasma-tissue concordance was 92% A subset of pts with longer PFS on treatment with ivosidenib had plasma *IDH1* mut clearance	[144]
*KRAS*	PDAC	Prospective	78 pts	ddPCR	Longitudinal ctDNA *KRAS* status was prognostic and predictive of responses to chemotherapy	[145]
*KRAS*	PDAC	Prospective	194 pts	ddPCR	*KRAS* plasma-tissue concordance was over 95% ctDNA detection was prognostic for survival	[146]
Multiple genes	PDAC	Prospective	77 pts	Guardant 360 NGS	Baseline ctDNA mutations included *TP53* (12.6%), *KRAS* (9.7%), *MET* (6.8%), *ARID1A* (4.8%), *NF1* (4.8%), and others < 3% ctDNA levels of *TP53* and *KRAS* were associated with radiographic responses New *TP53* subclonal variant mutations were the most common resistance mutations in progressions (75%)	[147]
Multiple genes (mostly *KRAS*)	PDAC	Systematic review and meta-analysis	2326 pts	various	ctDNA muts and high concentrations of ctDNA are prognostic for survival (PFS and OS)	[148]

Amp, amplification; BEAM, beads, emulsion, amplification, and magnetics; CCA, cholangiocarcinoma; CRC, colorectal cancer; ctDNA, circulating tumor DNA; DCR, disease control rate; ddPCR, digital droplet polymerase chain reaction; EGFRi, EGFR inhibitor; MAF, mean allele frequency; mCRC, metastatic colorectal cancer; mGC, metastatic gastric cancer; mGEC, metastatic gastric and esophageal cancer; mo, months; mut, mutated; NGS, next-generation sequencing; ORR, overall response rate; (m)OS, (median) overall survival; PDAC, pancreatic ductal adenocarcinoma; PDX, patient-derived xenograft; (m)PFS, (median) progression-free survival; PD, progression of disease; SD, stable disease; T-DXd, trastuzumab deruxtecan-nxki; VAF, variant allele frequency; WT, wild-type

Taking anti-EGFR therapy as an example [125–127, 131], ctDNA studies demonstrate that *RAS/EGFR* mutant clones emerge during treatment, which might regress upon the withdrawal of anti-EGFR therapy, thereby allowing for rechallenge with the targeted therapy [128]. This regression could not be reasonably assessed using tumor tissue because it would mean the risk of repeated biopsying. Being much less invasive, liquid biopsy and ctDNA analysis may allow for uncomplicated identification of patients suitable for rechallenge based on real-time genomic analysis. This idea was evaluated in the CHRONOS phase II trial, the first prospective interventional study to use liquid biopsy to guide anti-EGFR rechallenge therapy in CRC. Hence, liquid biopsy genotyping differentiated between patients with *RAS/BRAF/EGFR* mutated versus wild-type tumors, and one-third of wild-type patients had an objective response on rechallenge with an anti-EGFR antibody [129]. Other studies have identified specific *EGFR* and *RAS* mutations in the plasma after disease progression on treatment and highlight ctDNA as a tool in clinical practice to inform therapeutic development and tailor treatments based on emerging resistance mutations [128]. The feasibility of *BRAF* plasma testing and similar potential applications in this targetable gene have also been demonstrated [130, 131, 135].

Another good example of ctDNA application is in *HER2* amplified disease. Recent prospective trials have suggested that plasma *HER2* amplifications predict response to HER2-directed therapies such as lapatinib, trastuzumab, pertuzumab, and fam-trastuzumab deruxtecan-nxki (T-DXd) [136–139]. For example, better responses to T-DXd in gastric cancers were seen when plasma *HER2* and higher copy number amplifications were detected [138]. Also, changes in plasma copy number during HER2-directed therapy were associated with therapeutic response and survival in upper GI cancers and CRCs [122, 136, 137, 139]. Baseline and emerging resistance mutations detected in the plasma at the time of progression, such as *MYC*, *EGFR*, *FGFR2*, and *MET* amplification, have also been reported along with promising therapeutic strategies to overcome resistance, including combining anti-HER2 and other targeted or immune therapies [136, 139].

ctDNA can detect plasma *FGFR2* alterations, sometimes at a higher frequency than tissue testing, identify patients who may benefit from infigratinib, and identify emerging resistance point mutations [140–142]. Recently, plasma-tissue accuracy and survival data have also been described in CCA patients with *IDH1* plasma mutations treated with ivosidenib [144]. PDAC is highly *KRAS*-mutated and harbors rarer targetable mutations. Many studies have evaluated *KRAS* and *TP53* ctDNA detection and kinetics as prognostic and predictive biomarkers in PDAC cases [145, 147, 149, 150].

Early data suggest that plasma MSS and TMB have potential roles as prognostic and predictive biomarkers in GI cancers, although further study is needed to validate these assays in the guidance of immunotherapy [151, 152]. Collectively, these data highlight the evolving potential of blood-based molecular profiling to precisely guide patient therapy and overcome tumor resistance using only minimally invasive procedures. Consequently, clinical trials across GI cancers are increasingly focused on genomic testing of liquid biopsies in place of or in conjunction with tumor tissue biopsies to enroll patients and study their outcomes [64, 153, 154].

### Limitations

The liquid biopsy has been rapidly integrated into clinical practice for many solid tumors, but physicians should exercise caution when interpreting results. Tumor samples provide an abundance of tumor DNA compared to a liquid biopsy; therefore, liquid-based NGS may be limited by apparent lower sensitivity due to significantly lower levels of ctDNA [119]. Often, performing NGS on several genes means reduced depth of sequencing and sensitivity, not to mention added cost and effort compared to targeted sequencing of one or a few genes [119, 120, 155].

Studies have demonstrated that ctDNA levels and concordance may vary based on tumor histology, anatomic location, and stage [110, 156, 157]. Limitations arise if the tumor is a poor ctDNA shedder [122, 158]. Additionally, the presence of clonal hematopoiesis may lead to false positives in genotyping [159]. Unique molecular identifiers, dual barcode indexing, methylation assays, matched normal WBC DNA analysis, and cfDNA fragment length analysis can improve the accuracy of ctDNA analysis [109, 160, 161]; however, differences between laboratory testing platforms can contribute to discordant results across many mutations [162]. Despite the intrinsic limitations of assays, blood-based genomic profiling remains a promising tool for patient diagnosis, therapy guidance, and identification of patients for trial enrollment.

### ctDNA use in screening and diagnosing cases with insufficient tissue

As molecular profiling technology has advanced, there is increasing interest in using ctDNA for cancer screening (Fig. 2), diagnosis of inaccessible tumors, and management of cancers of unknown primary (CUP). Massive genomic profiling efforts, including The Cancer Genome Atlas’s Pan-Cancer initiative and other massive genomic profiling efforts, have identified tumor DNA, RNA, and protein patterns based on histology, anatomic location, and tissue types. These patterns can, in turn, be used to characterize undifferentiated tumors and identify tissues of origin [163]. For example, the highly-specific CancerSeek test [164] used ctDNA and protein biomarkers coupled with machine-learning to diagnose 8 early-stage cancers, including liver, gastric, pancreatic, esophageal, and colorectal cancer. The Circulating Cell-free Genome Atlas (CCGA) study [165] used a methylation-based assay from Grail to allow deeper sequencing and identified over 50 early-stage cancers, including liver/bile duct, gastric, pancreatic, esophageal, colorectal cancer, and anal cancer. The CancerSeek assay demonstrated sensitivities ranging from 69 to 98% in the detection of 5 cancer types, including liver, stomach, pancreatic, and esophageal [164]. The CCGA assay had 67.3% (CI 60.7–73.3%) sensitivity in 12 cancer types, including anal, colorectal, esophageal, liver/bile duct, pancreatic, and stomach [165]. Both assays had greater than 99% specificity, identifying the tissue of tumor origin with great accuracy. In another active study, Grail’s cfDNA methylation assay predicted the tissue of tumor origin with 92.3% accuracy [166]. Although these platforms are promising, they are still limited by inadequate sensitivity in early-stage diseases. Moreover, questions about disease management after detection, feasibility, ethics of general population testing, and cost–benefit ratios remain [167].

As it currently stands, GI cancer-specific but not yet multicancer screening tests have been FDA approved. Epigenomics Epi proColon® detects methylated SEPT9 DNA in the blood and was FDA approved on April 13, 2016, as the first blood-based CRC screening tool [168, 169]. Guardant’s LUNAR-2 ctDNA screening test for CRC is also currently under investigation, among others [170]. Blood-based hydroxymethylation and protein glycosylation signatures are promising biomarkers for the early detection of PDAC [171, 172]. Distinct circulating miRNAs signatures in blood and bile might act as biomarkers to differentiate between biliary cancers and other benign hepatobiliary diseases [173, 174]. However, miRNAs are nonspecific, and the best source of miRNA collection and the translation of miRNA assays into clinical practice are as yet undefined [175, 176].

Finally, the feasibility and utility of analyzing ctDNA from blood to characterize CUPs and identify targetable mutations have been described in multiple studies over the past decade [177–180]. Historically, patients with CUP had limited treatment options and poor prognoses because many standard-of-care therapies are tumor-specific. As more biomarkers are identified, broad blood-based NGS can uncover targetable mutations and identify more previously non-indicated therapies for these patients.

### ctDNA in MRD

Efforts are underway to identify patients at high risk of early relapse and develop interventions to lengthen patient survival. The strategy of MRD monitoring (Fig. 2) and eradication is already established in hematologic malignancies and is stimulating interest in GI cancers [181–183].

MRD ctDNA assays are often characterized as tumor-agnostic or tumor-informed. Tumor-agnostic approaches do not require pre-existing knowledge of a tumor’s genomic profile and often employ broad-based NGS, narrower PCR, or methylation assays to identify common cancer markers circulating in the blood. Numerous studies in localized or oligometastatic CRC patients undergoing curative surgery have demonstrated that ctDNA detection post-surgery or post-adjuvant therapy is a strong independent prognostic marker for survival [184–186]. Others suggest that ctDNA levels correlate with tumor burden and response to treatment [187]. Promising results in PDAC (often targeting *KRAS*) and gastroesophageal cancer have also been reported [188–191]. While limited by decreased sensitivity, tumor-agnostic methods are advantageous in their quick turnaround time, low cost, and simultaneous broad-based genomic profiling and resistance mechanism identification potential.

Although slower and more expensive, a tumor-informed approach offers higher sensitivity and is particularly attractive in assessing MRD when the ctDNA level is low, as in early-stage disease. Here, a patient’s tumor tissue is tested using genomic sequencing, and tumor-specific mutations are identified. These mutations are subsequently targeted in the blood using a personalized assay. Examples include SafeSeqS, CAPP-Seq, Archer DX, Radar, and Signatera. To date, most GI cancer studies have been in CRC and demonstrate sensitivities ranging from 48 to 100%, specificities ranging from 90 to 100%, positive predictive values (PPV) over 98%, and median lead times to radiographic relapse of about 8–9 months [192–203]. For example, using the Signatera test, a ctDNA positive status after adjuvant therapy and on postoperative longitudinal testing was found to confer 18 times and 30 to 40 times, respectively, higher risk of relapse compared to a ctDNA negative status. Moreover, ctDNA was found to outperform CEA in predicting relapse [192, 193, 198]. Studies characterizing ctDNA in the provision of early prognostic data are also emerging in gastroesophageal cancer, PDAC, and CCA [188, 189, 204, 205]. Some studies in the adjuvant and metastatic setting also suggest that ctDNA clearance or kinetics may predict treatment response and survival [198, 206].

We know that ctDNA positivity is highly prognostic but still lack knowledge on optimal disease management strategies for patients with MRD. While a positive test may theoretically hasten surveillance diagnostics, the role of local or systemic therapy escalation, in this case, is unclear, especially in the setting of serial, low-level ctDNA without evidence of radiographic relapse. Also, despite the achievable 0.01% level of ctDNA detection using tumor-informed assays, false negatives due to ctDNA levels being below the limit of detection should be considered when interpreting negative results. This is likely to be the case for low-shedding tumors, for example [207]. Therefore, in the absence of prospective, randomized data to support de-escalation strategies, a patient with a negative test should receive standard adjuvant therapy if not otherwise contraindicated. It is also important to realize that each company’s tests are uniquely constructed, using different error-correcting techniques that are frequently updated.

### Circulating tumor cells (CTCs)

It has been shown that CTCs are an intermediate stage of cancer metastasis. Like ctDNA, CTCs are obtained from peripheral blood; however, CTCs may have a greater clinical impact as they can be grown, propagated, and extensively studied in vitro and in vivo under optimal conditions [208, 209]. However, it is unclear if a single cell assay accurately reflects entire tumor heterogeneity.

Data supporting CTC enumeration as a predictor of clinical outcome dates back as early as 2004, when it was shown that patients with metastatic breast cancer had shorter mPFS and mOS if they had higher CTC levels [210]. Since then, this finding has been further validated across a wide range of tumor types, including GI cancers [208]. CTC enumeration shows promise in clinical management guidance; for example, as shown in breast cancer cases, the discovery of discordant driver mutations between an individual’s CTCs and their primary tumor may inform targeted treatment decisions [211–213].

Several commercial systems and clinical services (Epic, RareCyte™, CytoTrack, SRI FASTcell™) exist [214]. Current methods of CTC detection rely on one of three basic principles. The CellSearch® system was the first and is the only FDA-approved device for CTC enumeration [215]. The CellSearch® platform relies on antibody detection of CTC markers [208, 216]. Cohen et al. used the CellSearch™ system to estimate CTCs in their prospective multicenter mCRC study [217]. Their results showed that patients with ≥ 3 CTCs/7.5 mL blood had shorter mOS than patients with < 3 CTCs (*P* < 0.0001), and these differences persisted at follow-up time points after therapy. It was concluded that the number of CTCs was an independent predictor of disease-free survival (DFS) and OS in metastatic cancer [217]. An alternative CTC enumeration approach relies on isolating CTCs according to prespecified cancer-specific gene products (RNAs and proteins) [208, 218]. However, this method involves the lysing of captured CTCs, limiting their use in downstream analyses. A third technique isolates CTCs according to their physical characteristics; CTCs are generally much larger than blood cells (30 µm vs. 7–9 µm), allowing their isolation and enumeration [219].

## Future directions

### CTCs and organoids

CTC study has already added to our understanding of cancer metastases. For example, it has been described that CTCs often carry genetic variations in driver mutations that are different from the primary tumor; these differences would likely help explain the propensity for primary tumors to metastasize and seed into other organs [220].

As the next frontier in precision medicine, the ability to grow and expand CTCs ex vivo is an invaluable, noninvasive tool in the study of cancer biology and metastasis [208, 209, 216]. One step further, the ability to create CTC-derived xenografts by injecting CTCs directly into immunocompromised mouse hosts holds vast implications in both research and clinical settings. These organoids maintain tumor heterogeneity and allow investigation of therapeutic elements on the xenograft that mirror patient response to the same treatment [221, 222]. Such clinical applications have already been used to perform in vivo drug screens with high success and hold implications for new drug discovery [221]. For research purposes, organoids can be used for disease modeling to understand the process of carcinogenesis. They can be manipulated easily using retroviruses and inhibitors, for example, and can be used to identify key driver mutations, as shown already in some GI cancer organoid studies [223, 224]. The clinical role of CTCs is currently limited but is expected to expand on the heels of technology improvements, including CTC-isolation and organoid-development techniques.

### What can we learn from the blood, and how can we use biomarker testing in the future?

Tissue molecular profiling provides clinically significant subtyping of all GI cancers. The liquid biopsy promises dynamic tumor characterization through various platforms, and we believe these capabilities will be increasingly incorporated into clinical cancer management. The liquid biopsy is already integrated into the standard of care for gastric, esophageal, and GEJ cancers, for which NCCN guidelines recommend plasma ctDNA profiling by NGS to detect targetable alterations or clones with altered treatment sensitivity when patients are not candidates for tumor-tissue biopsy and NGS [207].

In the future, molecular profiling of the liquid biopsy will likely complement or replace the GI tumor-tissue biopsy in select scenarios. Future therapeutic studies should include ctDNA analyses to identify prognostic and predictive liquid biopsy biomarkers. Serial testing should also be further assessed as a way to quickly and noninvasively characterize disease response or mechanisms of resistance. Finally, “MRD with no evidence of radiographic disease” might become a theoretical “new stage,” warranting novel treatment strategies. As liquid biopsy techniques improve, blood-based testing will hopefully better identify MRD and screen early-stage GI tumors with hopes of curing more patients and improving outcomes.

## Summary

Molecular profiling for patients with GI malignancies is clearly making an impact and has become the standard of care in many situations. In fact, in 2021, ASCO chose molecular profiling in GI cancers as its Advance[ment] of the Year [225]. An increasing number of actionable biomarkers are being identified, and drugs that act on these biomarkers are continually being developed, providing patients with better treatment options, improved quality of life, and increased survival compared to standard therapy alone. Likewise, analytical methods using tumor tissue and, more recently, blood are constantly being developed and improved, promoting the identification of biomarkers and gene signatures that help diagnose disease and predict therapy success in this oftentimes refractory group of malignancies. Together with machine learning, our evolving biomarker technology is promising to help us fight an even smarter war against GI cancers.

## Data Availability

Not applicable.
